# Dementia Knowledge Among Primary Healthcare Physicians in Riyadh, Saudi Arabia

**DOI:** 10.7759/cureus.61112

**Published:** 2024-05-26

**Authors:** Zeyad A Alsalem, Nawaf M Alghathber, Faris S Alowain, Muath S Alqahtani, Nouf G Alharbi

**Affiliations:** 1 Family Medicine, King Fahad Medical City, Riyadh, SAU

**Keywords:** riyadh, saudi arabia, dementia, primary health care, knowledge, memory disorders, elderly patients, cognitive disorders, early-onset dementia, alzheimer’s dementia

## Abstract

Introduction: Dementia poses a significant healthcare challenge globally, and healthcare providers must have adequate knowledge about its diagnosis, management, and support services. By assessing the knowledge level of primary care physicians in Riyadh, we can identify potential gaps and areas for improvement in dementia care, ultimately enhancing patient outcomes and quality of life. This study holds promise in shedding light on the current state of dementia knowledge among primary healthcare physicians in Riyadh and offering insights into strategies to enhance dementia care in this region.

Methods: This cross-sectional questionnaire-based study was conducted from the first of June 2023 to the end of December 2023 in Riyadh, Saudi Arabia. A validated questionnaire was used to assess physicians’ knowledge, attitude, and practice toward dementia.

Results: A total of 151 physicians completed the questionnaires. The majority were male (55%), below 30 years of age (88.1%), and family medicine residents (84.8%). Most (74.8%) recognized old age as the most significant risk factor; an overwhelming majority of participants (98.7%) could not identify the minimum course of treatment to judge a medication’s effectiveness. The average score of correct responses (7.74 ∓ 4.11) was equivalent to 38.7%. Furthermore, the average correct responses were significantly different among the different job levels of the participants.

Conclusions: The findings of this study highlight a lack of knowledge among primary care physicians regarding dementia, emphasizing the crucial importance of physician education in this area. Additionally, the results strongly indicate the need for emphasis on dementia education within the undergraduate medical curriculum, family medicine curriculum, and physician training programs. By addressing these educational gaps, we can better equip physicians to provide optimal care and support for individuals with dementia, ultimately improving patient care and quality of life.

## Introduction

Dementia is a significant contributor to debilitating health outcomes and impaired independence [1]. In 2018, it was estimated that around 47 million people worldwide have dementia, and by 2050, the number is projected to triple to 131 million [2]. According to the WHO, the Middle Eastern region is witnessing an alarming increase in dementia, with the prevalence projected to increase by 125% by 2050 [3]. In Saudi Arabia, the number of older people is increasing. About 6.5% were over 60 years old in 2016. By 2050, the number will reach 10 million, representing 25% of the total population [4]. Early diagnosis facilitates providing high-quality care for the patient and their caregiver. Studies show that early detection minimizes the psychological stress on patients and their relatives [5].

Primary care physicians play an essential role in detecting and diagnosing dementia in the elderly. They are frequently the first point of contact with older people, so they need a high index of suspicion when encountering patients with memory complaints [6]. Limitations in healthcare providers' attitudes, knowledge, and communication between patients and their caregivers are significant contributors to missed and late detection and diagnosis [7]. It significantly impacts patients, their families, the healthcare system, and the economy. The Boettiger et al. study projects increasing healthcare costs due to aging and non-communicable diseases in Saudi Arabia through 2030 [8]. In addition, dementia can impair function, limit daily activities and self-care routines, and increase caregiver burden [7,9].

The prevalence of various forms of dementia in the Middle East and North Africa (MENA) region is substantial; however, there is a shortage of research evaluating the knowledge, attitude, and practice (KAP) of primary healthcare physicians about dementia [4]. A cross-sectional study of six primary healthcare centers from Riyadh City, Saudi Arabia, estimated the prevalence of dementia to be 11% to 16% of study participants [10]. In another cross-sectional study from Saudi Arabia, the prevalence of dementia was determined to be 6.4% in individuals aged 60 years and above [4]. Similar findings were reported in a systematic review that estimated the incidence of dementia ranging from 1.1% to 2.3% for individuals aged 50 years and above and from 13.5% to 18.5% for those aged 80 years and above [3]. In our literature search, we could not find any relevant study on the KAP of physicians regarding dementia from Saudi Arabia. However, one study investigated the KAP of medical students in Saudi Arabia. Aldharman et al. reported that the majority of the participants demonstrated a poor level of knowledge about dementia. The mean knowledge score was 13.68 ± 3.18 (on a scale of 25) [11].

Moreover, numerous studies around the world have shown that physicians possess a limited to moderate understanding of dementia [5,9]. According to Steiner et al., a disparity was observed in the comprehension of the diagnostic criteria for dementia among physicians compared to their expertise in the epidemiology and management of the condition in Brazil [9]. A study conducted by Leung et al. in Hong Kong analyzed the self-reported proficiency levels of the participants and found that a substantial number of the subjects reported inadequate expertise on dementia due to a lack of practical experience. Despite this shortfall, the participants demonstrated a favorable attitude toward the early detection of dementia [5]. A study from the United States revealed that physicians who possessed knowledge of the Physician's Guide to Assessing and Counseling Older Drivers showed adequate knowledge of dementia [12]. However, another study with 22 participating physicians showed a deficit in comprehension of the dissimilarity between mild cognitive impairment (MCI) and dementia.

Furthermore, disparities in physician techniques to diagnose MCI in older adults were also seen [13]. A low KAP of physicians can have substantial consequences for the patients. The results of a recent review demonstrated that a prolonged time of detection and diagnosis can lead to adverse social and clinical outcomes for individuals with Alzheimer's disease-related dementia [7].

In a study by Lo et al. in Macao, the mean score for dementia care knowledge was 87.02 ± 14.01 among primary healthcare professionals. Additionally, the mean score for attitude was 69.52 ± 5.83, while the mean score for preventive practice was 77.88 ± 13.18. The results indicated a significant difference in preventive practice between doctors (79.89 ± 13.77) and nurses (75.91 ± 12.33) (p-value = 0.023) [2]. The General Practitioner Attitudes and Confidence Scale for Dementia (GPACS-D) is a psychometrically sound instrument for evaluating general practitioners' confidence levels and attitudes toward dementia. This scale comprises 15 items and consists of three subscales [14]. A study from Malaysia utilizing GPACS-D reported a mean confidence score of 2.96 (±0.76) among the participants in managing dementia. The study's results revealed that several factors, including dementia knowledge, higher scores for attitudes toward dementia, and prior education on dementia, were significantly associated with higher levels of confidence in managing dementia [6].

Similarly, Khait et al. investigated the KAP of dementia among students. They reported that the mean score of the students was 24.53 ± 7.81 out of a total of 48 (52%). The lowest scores were obtained in the communication and behavioral aspects of individuals with dementia and in the realm of risk factors and health promotion in dementia care [15].

In summary, dementia is one of the significant disabling conditions for the elderly worldwide. Diagnosis of dementia aids in slowing down its progression, provides support for patients and their families, and decreases the burden on the healthcare system. Accessibility to primary healthcare centers (PHCs) puts the primary physicians on the frontlines for detecting and managing such patients. Therefore, evaluating the KAP of primary care physicians will set the stone for bridging the gaps in knowledge and improving the quality of care.

## Materials and methods

Study design

This cross-sectional questionnaire-based study was conducted from the first of June 2023 to the end of December 2023 in Riyadh, Saudi Arabia. A validated questionnaire was used to assess physicians’ KAP toward dementia.

Questionnaires

In this study, a self-administration questionnaire was used. The questionnaire was developed from previous studies after an in-depth literature review. The questionnaire is written in English and includes 25 multiple-choice questions. It consists of two main parts - "Part 1: Sociodemographic and Professional Characteristics," which includes age, gender, city, professional qualification, and working area, and "Part 2: Knowledge Regarding Dementia," which consists of 20 multiple-choice questions. Questions were adapted from the study by Pentzek et al., and permission was obtained from the authors [16]. All questions were included in the questionnaire without any changes.

Participants

The target of our study was primary care physicians, including family physicians at all levels and general practitioners working in PHCs. No names were announced, and their information was treated with high confidentiality. The participants were informed of their right to withdraw at any point through a statement on the questionnaire cover page. A total of 151 participants, with physicians of various levels, participated in this study.

Data collection

The study was conducted through questionnaire distribution. Emails, social media messages, and on-site visits were used to invite the participants to join in the study.

Data analysis

Data were placed on an Excel sheet (version 15.39, MacOs Sierra 10.12.6) and were analyzed using SPSS (version 23.0, IBM Corp., Armonk, NY) statistical software. Descriptive statistics were used for the frequency, percentage, correlation, and p-value. A p-value less than 0.05 was considered statistically significant. Independent t-tests and one-way ANOVA were applied to sociodemographic variables.

Ethical approval

The institutional review board of King Fahad Medical City provided ethical approval for the study. Regarding consent, the following statement was included: “By completing this questionnaire, you are giving us permission to use the information provided in the study.” All information given is treated as confidential. Participants have the right to withdraw from the study at any time. Also, participants with questions about the study could contact the principal investigator or the research team.

## Results

This study enrolled 151 participants. More than half were males (55%), with an age category of less than 30 years (88.1%). Concerning job level, most were family medicine residents (84.8%), and the proportion of first-year, second-year, and third-year residents were 29.8%, 27.8%, and 27.2%, respectively. Most participants reported working in primary healthcare centers (Table 1).

**Table 1:** Sample characteristics (N = 151)

Characteristics	Family medicine, N = 151, n (%)
Sex	
Male	83 (55.0)
Female	68 (45.0)
Age	
<30 years	133 (88.1)
31-35 years	9 (6.0)
36-40 years	3 (2.0)
>40 years	6 (4.0)
Job level	
General practitioner	4 (2.6)
Resident first-year family medicine	45 (29.8)
Resident second-year family medicine	42 (27.8)
Resident third-year family medicine	41 (27.2)
Registrar	3 (2.0)
Senior registrar	8 (5.3)
Acting consultant	2 (1.3)
Consultant	6 (4.0)
Working area	
Primary healthcare center	108 (71.5)
Secondary hospital	4 (2.6)
Tertiary hospital	39 (25.8)

Table 2 shows participants' responses to the knowledge questions about dementia. Regarding the correct response rates, the three highest were for item 10 (74.8%), which assessed the knowledge about the most significant risk factor for developing Alzheimer's disease. Item 20 (71.5%) presented a scenario about a patient developing sudden confusion and disorientation and asked participants to identify delirium correctly. Additionally, item 2 (70.9%) assessed the participants' ability to identify acetylcholinesterase inhibitors as the preferred drug treatment for mild Alzheimer's.

**Table 2:** Participants’ responses to the knowledge questions about dementia (N = 151) Chi-squared or Fisher’s exact test was used. * Statistically significant at α ≤ 0.05, ** statistically significant at α ≤ 0.01, and *** statistically significant at α < 0.001.

Questions (correct response and three distractors, separated selection correct by semicolon)	Wrong responses (%)	Correct responses (%)	"I don’t know" responses (%)	P-value
1. When someone with Alzheimer’s disease shows frequent lip-smacking movements and grimacing, one should suspect an adverse reaction from which group of psychotropics? (Butyrophenones; distractors: barbiturates; selective serotonin reuptake inhibitors (SSRI); monoaminoxidase inhibitors)	36 (23.8)	55 (36.4)	60 (39.7)	<0.041*
2. In the current climate of knowledge, the preferred drug/drugs for the treatment of mild Alzheimer’s disease is/are ... (Acetylcholinesterase inhibitors; distractors: estrogen; acetylsalicylic acid; vitamin E).	10 (6.6)	107 (70.9)	34 (22.5)	<0.001***
3. The wife of a 64-year-old patient reports that her husband’s cognition severely declined over the last two months. He often forgets about things, lacks concentration, and loses interest easily. At the patient’s examination, impaired vision and myoclonia (spontaneous convulsions) are evident. Which diagnosis most closely matches the portrayed symptoms? (Creutzfeldt-Jakob disease; distractors: vascular dementia; Pick’s disease; Huntington’s chorea)	78 (51.7)	18 (11.9)	15 (36.4)	<0.001***
4. The score of the Mini-mental State Examination (MMSE) ranges from 0 to 30. Which of the following statements is true? (A score of 14 is an obvious sign of dementia; distractors: a score of 23 rules out dementia; higher scores are a sign of dementia; patients in the early stages of dementia frequently attain scores above 27.)	28 (18.5)	65 (43.0)	58 (38.4)	<0.001***
5. Which two additional symptoms (besides dementia) point to the presence of a (potentially reversible) normal pressure hydrocephalus? (Walking disturbances and incontinence; distractors: hallucination and tremor; hyperorality and hypersexuality; depression and social isolation)	32 (20.5)	70 (46.4)	50 (33.1)	0.001**
6. Which statement about the relation of dementia to depression is NOT true? (Depression usually occurs in the late stages of Alzheimer’s disease; distractors: depressive symptoms frequently occur in the early stages of Alzheimer’s disease; cognitive symptoms in depressive patients can resemble symptoms of Alzheimer’s disease; a depressive disorder and Alzheimer’s disease can exist in parallel)	77 (51.0)	30 (19.9)	44 (29.1)	<0.001***
7. All of the following are potentially treatable etiologies of dementia except ... (Pick’s disease; distractors: Addison’s anemia; subdural hematoma; normal pressure hydrocephalus)	23 (15.2)	84 (55.6)	44 (29.1)	<0.001***
8. Which of the following methods is the most appropriate for a person with moderate to severe dementia? (Reminiscence therapy/validation; distractors: memory training; reality orientation; informing about the clinical picture of dementia)	70 (46.4)	18 (11.9)	63 (41.7)	<0.001***
9. How long is the minimum course of treatment using an anti-dementia drug (compatibility assumed) in order to judge its effectiveness? (3 months; distractors: 1 month; 18 months, 24 months	63 (41.7)	2 (1.3)	86 (57.0)	<0.001***
10. What is the most significant factor in the risk of developing Alzheimer’s disease? (Old age; distractors: arteriosclerosis; malnutrition; strong exposure to aluminum)	20 (13.2)	113 (74.8)	18 (11.9)	<0.001***
11. Assuming that you have 100 patients aged over 85 (excluding nursing home patients), how many dementia sufferers would you expect to find among them? (25 to 35; distractors: 5 to 15; 40 to 50; 60 to 70)	75 (49.7)	29 (19.2)	47 (31.1)	<0.001***
12. Which diagnostic result or information taken from the patient’s history is rather atypical for Alzheimer’s disease? (Abruptly incipient symptoms; distractors: normal laboratory values (serum); depressive symptoms; age-appropriate EEG)	34 (22.5)	77 (51.0)	40 (26.5)	<0.001***
13. A frontotemporal dementia is usually characterized by ... (Inadequate and rude social behavior early in the course of the disease; distractors: visuoconstructional deficits (clock drawing) early in the course of the disease; impairment of short-term memory early in the course of the disease; acoustic hallucinations early in the course of the disease)	33 (21.9)	89 (58.9)	29 (19.2)	<0.001***
14. Which of the following recommendations is usually appropriate to a moderately demented person? (The patient should maintain a constant daily routine with simple tasks; distractors: the patient should have a high variety in his/her daily activities (e.g., day trips, cultural activities) to distract him/her from his/her disease; the patient should be advised of appointments (e.g., barber, festivities) early (several days in advance); the relatives should train the patient’s memory by repeatedly asking for the current date and recent events.)	29 (19.2)	62 (41.1)	60 (39.7)	<0.001**
15. Which statement is correct regarding Alzheimer’s patients’ fitness to drive? (Fitness to drive is mostly preserved only in mild disease stages; distractors: fitness to drive can be assessed with the MMSE – a score of 25 or lower fitness to drive is not ensured in most cases; fitness to drive is mostly preserved in moderate dementia stages; fitness to drive is mostly not ensured already in patients with very mild cognitive impairment.)	36 (23.8)	28 (18.5)	87 (57.6)	<0.001***
16. Which of the following examinations is normally LEAST relevant to the diagnostic evaluation of dementia? (Protein electrophoresis; distractors: thyroid-stimulating hormone (TSH) level; electrolyte level; vitamin B and folic acid level)	47 (31.1)	57 (37.7)	47 (31.1)	<0.516
17. Which statement concerning the Mini-mental State Examination (MMSE) is true? (It lacks accuracy in recognizing the initial stages of dementia; distractors: most tasks check memory function; it does not comprise orientation tasks; it can only be conducted by specialists.)	37 (24.5)	42 (27.8)	72 (47.7)	<0.001**
18. What is the most frequent cause of a significant and increasing impairment of short- and long-term memory in people aged over 65? (Alzheimer’s disease; distractors: Parkinson’s disease; normal aging; vascular dementia)	53 (35.1)	62 (41.1)	36 (23.8)	<0.031*
19. Which statement is true concerning the treatment of Alzheimer’s patients with mild to moderate depressive symptoms? (Treatment of depressive symptoms should ideally be carried out with selective serotonin reuptake inhibitors (SSRI), anti-dementia drugs can be given simultaneously; distractors: depressive symptoms should not be treated because antidepressive therapy is always accompanied by worsening of cognitive symptoms; treatment of depressive symptoms should ideally be carried out with tricyclic antidepressants, anti-dementia drugs can be given simultaneously; already taken anti-dementia drugs should be discontinued, and benzodiazepines should be given.)	17 (11.3)	52 (34.4)	82 (54.3)	<0.001***
20. A patient you have not met for a long time develops confusion and disorientation according to his relatives within two weeks, and he cannot maintain alertness. Which diagnosis is the most likely? (Delirium; distractors: Alzheimer’s disease; depression; Pick’s disease)	6 (4.0)	108 (71.5)	37 (24.5)	<0.001***

In contrast, the three lowest correct responses were for item 9 (1.3%), which addressed the minimum course of treatment using an anti-dementia drug to judge its effectiveness. Meanwhile, both item 3 and item 8 had 11.9% correct responses. Item 3 described an elderly patient with rapidly declining cognition, forgetfulness, impaired vision, and myoclonia, and queried the participant to identify Creutzfeldt-Jakob disease. Item 8 examined participants' ability to specify the most appropriate method for managing a person with moderate to severe dementia, with reminiscence therapy/validation as the correct answer. The classification of participants' responses as correct, wrong, and "I don't know" significantly differed across 19 items except for item number 16 (Which of the following examinations is normally least relevant to the diagnostic evaluation of dementia?) (Table 2).

Table 3 demonstrates the participants' average number of correct responses. The average score for the number of correct responses was 7.74, with a standard deviation of 4.11. This score indicates that the participants' correct responses were in the lower arm (7.74 out of 20), which means the average correct response was 38.7%.

**Table 3:** Participants’ average number of correct responses (N = 151)

Items	Mean ± SD
Number of correct responses	7.74 ± 4.11

Tables 4, 5 compare the average of correct responses across the participant's demographics. Males and females had similar averages of correct responses of knowledge about dementia. Also, the average correct responses of knowledge about dementia did not significantly differ based on the working area of the participants. However, the average of correct responses about dementia was found to be significantly different across the job level of the participants (F (7, 143) = 3.868, p = 0.001).

**Table 4:** Independent t-test results for sociodemographic variables concerning participants’ average number of correct responses about dementia (N = 151)

Outcome variables	Independent variables		Mean ± SD	df	T-value	P-value
	n	Gender				
Number of correct response	83	Male	8.01 ± 4.23	149	0.915	0.362
	68	Female	7.40 ± 3.96			

**Table 5:** One-way ANOVA results for sociodemographic variables concerning participants’ average number of correct responses about dementia (N = 151) ** Statistically significant at (α ≤ 0.01).

Outcome variables	Independent variables		Mean ± SD	df	F-value	P-value
	n	Job level				
Number of correct response	4	General practitioner	8.00 ± 1.41	7, 143	3.868	0.001**
	45	Resident first-year family medicine	7.44 ± 3.21			
	42	Resident second-year family medicine	6.52 ± 4.40			
	41	Resident third-year family medicine	7.59 ± 4.17			
	3	Registrar	11.00 ± 3.61			
	8	Senior registrar	10.38 ± 3.34			
	2	Acting consultant	7.50 ± 2.12			
	6	Consultant	14.17 ± 3.82			
	n	Working area	Mean ± SD	2, 148	1.620	0.201
	108	Primary healthcare center	7.66 ± 3.89			
	4	Secondary hospital	4.50 ± 4.51			
	39	Tertiary hospital	8.28 ± 4.58			

## Discussion

Dementia is a growing public health concern, with the Middle Eastern region projected to witness an increase in prevalence [3]. Additionally, in Saudi Arabia, the aging population is expected to contribute to a significant increase in the number of individuals with dementia [4]. Knowledge and early identification of dementia are crucial as they can mitigate the psychological stress experienced by patients and their families [5]. Furthermore, the knowledge and attitudes of primary care physicians toward early detection play a critical role in the effectiveness of this approach [5].

Our study found an average correct response rate of only 38.7% among primary healthcare physicians in Riyadh, Saudi Arabia, regarding their knowledge of dementia. For example, participants demonstrated a good understanding of dementia risk factors, treatment for mild Alzheimer's, and delirium diagnosis. On the other hand, they exhibited significant knowledge gaps in areas like minimum anti-dementia drug treatment duration, diagnosis of Creutzfeldt-Jakob disease, and appropriate therapy for moderate to severe dementia. The results show that the basics of the disease are well-known by primary care physicians. However, knowledge about the details of management and other differentials is limited.

The variability in knowledge across different content areas aligns with prior reports from other regions, highlighting inconsistencies in dementia expertise among healthcare professionals globally. Interestingly, in our study, gender and practice setting did not significantly influence knowledge scores. However, lower training levels correlated with poorer dementia knowledge, emphasizing the need for comprehensive education throughout undergraduate and postgraduate medical curricula.

Notably, our findings align with previous research from Saudi Arabia and nearby countries, collectively underscoring significant knowledge deficits related to dementia among healthcare professionals in the region. Aldharman et al. reported a mean knowledge score of only 13.68 out of 25 among health college students in Saudi Arabia [11]. Similarly, Al-Awad et al. found that while primary care physicians had a positive attitude toward dementia care, their mean knowledge score was a suboptimal 36.4 out of 50 [17]. Beyond Saudi Arabia, Paul et al. revealed moderate dementia knowledge (mean 5.3 out of 7) among healthcare professionals in Qatar, with a lack of awareness about recent pathophysiological advances [18]. Pham et al. also noted poor dementia knowledge and low confidence levels among primary care providers in Vietnam [19].

Dementia, a complex neurological condition, poses significant challenges for physicians in terms of knowledge gaps. The reasons behind these gaps are multifaceted, from various factors contributing to a lack of comprehensive understanding and expertise in this area. First, dementia itself is a multidimensional disorder with various underlying causes, subtypes, and clinical presentations [20]. Understanding the detailed pathophysiology, manifestations, and progression patterns of different dementia types can be challenging, which causes a lack of comprehensive understanding of such a condition. Additionally, as research advances, diagnostic criteria and guidelines for dementia are periodically updated, making it difficult for physicians to stay abreast of these changes and implement them effectively in clinical practice [21].

Furthermore, many medical schools and residency programs have traditionally placed limited emphasis on geriatric medicine, including dementia care [22]. This lack of focused training during the formative years of a physician's education can result in significant knowledge gaps throughout their careers. The variability in the clinical presentation of dementia compounds this issue, further complicating our understanding. Symptoms can range from cognitive impairment to behavioral and functional changes, which could overlap with other disorders, making it challenging for physicians to recognize and accurately diagnose the condition, especially in its early stages [23].

The field of dementia research is rapidly evolving, with continuous discoveries emerging related to risk factors, biomarkers, and potential treatments [21]. Keeping up with these advancements can be a formidable task for physicians juggling multiple clinical responsibilities and competing demands on their time and attention. Moreover, access to specialized training programs, continuing medical education opportunities, or resources focused on dementia care may be limited in certain regions, further exacerbating physician knowledge gaps [24].

Stigma and misconceptions surrounding dementia can also play a significant role in perpetuating knowledge deficits [25]. Some physicians may hold biases or outdated beliefs about the condition, hindering their willingness to learn more or seek appropriate training. Overcoming these profoundly ingrained perceptions and fostering a more inclusive and informed approach to dementia care is crucial.

The identified knowledge gaps have significant clinical implications for dementia care delivery in Saudi Arabia. The lack of understanding of key aspects, such as recognizing early symptoms and determining adequate therapeutic trial durations, can directly impede timely diagnosis and appropriate management [26]. Missed or delayed diagnosis prevents patients from accessing interventions during the initial window when treatments are most beneficial in potentially slowing symptom progression [27]. Suboptimal knowledge about effective pharmacotherapeutic strategies and monitoring parameters risks inappropriate medication use, compromises treatment response, and increases the likelihood of adverse events [28]. Furthermore, the variability in knowledge across different domains suggests an inconsistent approach to clinical evaluation, with some aspects like risk factor assessment receiving emphasis. At the same time, other crucial areas like differential diagnosis are relatively neglected.

A key strength of this study is the broad assessment spanning multiple knowledge domains using a validated instrument. Furthermore, the diverse participant sample across job levels and practice settings enhances the generalizability of the findings. However, the self-reported nature of the survey could introduce response biases. Additionally, the single-city sample limits the regional extrapolation of results. Future multicenter studies incorporating clinical vignettes, direct observation of skills, and longitudinal knowledge assessments would provide a more comprehensive evaluation of dementia knowledge and its translation into clinical practice.

Limitations

This study has multiple limitations that should be acknowledged. The majority of participants were young physicians (<30 years old), and most (88%) were resident physicians. The study only covered primary care physicians in Riyadh City. Non-primary healthcare physicians and specialties who assess and assist people with dementia were not included. Other cities and rural areas of the kingdom were not included. However, primary healthcare practice typically covers patients with dementia in Riyadh. The lack of studies about knowledge of dementia in our region has hindered our ability to establish a foundation of knowledge to anticipate the outcomes of this study.

## Conclusions

In conclusion, this study shows deficits in knowledge related to dementia among primary healthcare physicians in Riyadh. While the physicians demonstrated a satisfactory understanding of the fundamental aspects such as dementia risk factors, treatment for mild Alzheimer’s, and the diagnosis of delirium, critical weaknesses were identified in their knowledge of more nuanced management and differential diagnoses. These findings highlight the need for educational interventions to strengthen physicians' comprehensive knowledge of dementia, from risk factors and clinical presentation to diagnostic evaluation and treatment principles. Empowering physicians with comprehensive dementia knowledge is crucial for the country's ability to effectively address the growing burden of dementia.
